# Insight into age-related changes of the human facial skeleton based on medieval European osteological collection

**DOI:** 10.1038/s41598-023-47776-4

**Published:** 2023-11-23

**Authors:** Anna Walczak, Marta Krenz-Niedbała, Sylwia Łukasik

**Affiliations:** Institute of Human Biology and Evolution, Faculty of Biology, Adam Mickiewicz University in Poznań, Uniwersytetu Poznańskiego 6, 61-614 Poznan, Poland

**Keywords:** Anthropology, Biological anthropology

## Abstract

Aging changes in the facial skeleton are concentrated mostly in orbits, maxilla and mandible. The aim of this study was to analyze metric traits of the adult viscerocranium in a medieval sample from Cedynia (Poland, Central Europe) and confront the results with literature data for modern populations. It was assumed that diet-related greater biomechanical forces generated during mastication in medieval versus modern times led to slower rate of bone resorption with age. 3D models of the facial skeleton are created for 230 individuals, categorized into young, middle and old adults, and a subgroup of edentulous middle adults is distinguished. Orbits, piriform aperture, maxilla and mandible are measured using Geomagic Studio 12 and analyzed among age categories as well as dentate and edentulous subgroups. The values of the orbital and piriform aperture measurements tend to increase with age and reached statistical significance in males (right orbit height, left orbit width, piriform aperture surface area). In females, maxillary height significantly decrease at right first premolar and first molar, together with height of the right mandibular ramus. In edentulous individuals of both sexes the orbits are wider, and maxillary and mandibular heights are lower than in dentate individuals. This study reveals similar character and direction of the aging process of the facial skeleton in medieval and modern adults, however slower rate of resorptive changes is found in the former sample, which suggests, that diet-related biomechanical forces can influence intensification of the aging processes in the facial skeleton.

## Introduction

Facial aging is a complex, multifactorial process that leads to changes in ligaments, muscles, adipose tissue, skin, and bones^1^. It represents a transition from youth, when the bone morphology and soft tissue envelope volume stay in balance, to the elderly, when this balance gets disturbed^2^. Skin undergoes atrophy with the reduction of collagen and weakening of the elastin structure. Muscles can undergo hypo- or hypertonicity. Also, the loss of facial fat compartments can occur. Bone structures, after reaching their peak bone mass, gradually lose their volume and density^1^. Involutional changes progress in separate tissues and together lead to the appearance of an aged face.

A better understanding of the facial aging process has been an object of multiple studies, but most of them have focused on changes occurring in soft tissues and how to surgically correct them^3–5^. Thus, facial aging mainly has been analyzed within medical sciences and hard tissue alterations have not been considered. Recent research, has found that aging also affects facial bones, and those changes significantly contribute to the appearance of an aging face^6^. Since the facial skeleton is a scaffolding for other tissues, resorption and volume loss of midface elements reflect weakened skeletal support^7^. In consequence, the elements of the facial skeleton most severely affected by resorption correspond with the most visible signs of aging^8,9^.

Bone tissue, like any other tissue, has its own metabolism, which gets disturbed in aging process. In normal conditions bone metabolism is defined by the balanced relationship between bone resorption and bone formation. At different stages of life this relationship may take on different values, favoring one of those phenomena. Bone formation is more intense during childhood, while in adulthood both processes stay in balance, and in the elderly resorption starts to dominate^1,10^. We know that involutional bone loss in the craniofacial skeleton is uneven and site specific. Structures most severely affected are: superomedial and inferolateral parts of orbital rim, *piriform* area of maxilla and the prejowl region of the mandible. The explanation of the mechanism of changes occurring in the facial skeleton with age was proposed by Sharabi et al.^9^. They formulated a hypothesis about the significant role of mechanotransduction in facial skeleton aging, which is the process of intercellular transduction of a mechanical signal into the effector cells—osteoblasts^11^. With age facial muscles are getting weaker and that results in decrease of mechanical stimulation of bone tissue. This signal received by bone tissue cells results in favoring bone resorption over bone formation, in accordance with the Wolff’s law. Indeed, several studies revealed that with age muscle quality deteriorates^12,13^ and medical and experimental research found a relationship between changes in craniofacial complex and ablation of facial muscles and nerves^14,15^. Numerous studies specifically showed a correlation between mastication muscles parameters and craniofacial morphology, concentrating mainly on the relationship between chewing muscle features (volume and cross-sectional area) and facial height and width (e.g.^16–18^).

Mechanical loading has been repeatedly shown to play a crucial role in modelling the cortical and trabecular bone tissue^19^. Some authors suggested that certain parts of the facial skeleton are prone to resorption related to aging in result of the lack of biomechanical stress^9,20^, which is in accordance with mechanotransduction hypothesis. Mendelson and Wong^8^ suggest that regions most susceptible to resorption are more mobile areas during facial animation. They focused on the orbital area and noted that muscles of this region must have less ligamentous fixation of the soft tissues to the bone because of their intensive movement. Therefore, it would explain why selective resorption is present in orbital rims. William and Slice^20^ have claimed that changes in superomedial part of the orbital rim may be related to enlargement of the frontal sinuses^21^. Enlow^22^ proposed an idea, which became widely accepted, that the craniofacial skeleton has a tendency to expand or enlarge with age. Applying cephalometry, he examined growth of the facial skeleton from infancy to adulthood and noted an increase in face length, width and depth^8^. Numerous studies have reported this trend also in adults in the process of aging, and using different anthropometric measurements revealed that the skull continuously expands in horizontal and vertical dimensions^23–25^. Shaw and Khan^26^ challenged the idea that facial aging is a process of volume loss and skeletal atrophy, from analyzing aging changes in specific regions of the facial skeleton. Since the beginning of the twentieth century, selective resorption in some areas of the facial skeleton has been confirmed (e.g.^26–29^). Application of new methods and technologies have had a great impact on growing understanding of the aging process^8^. However, involutional changes in the facial skeleton are still not entirely recognized, partly because of contradictory findings in the studies published so far. The character of changes and even the presence of the processes are still being questioned. This situation may be caused by several factors, including the variety of methods, as well as different age ranges, diverse ethnic background and various sampling methods of the examined persons^30^.

Changes occurring with age in soft and hard tissues are influenced to a variable degree by muscular function, genetics and environmental factors^31–33^. Additionally, jaw resorption can be moderated by tooth loss^30^. Those factors differ in the past in comparison to modern times, especially biomechanical forces, which are related to the texture and consistency of food. The medieval diet was composed of much more coarse, abrasive and unrefined foods and required longer and more powerful chewing^34^. Nowadays people consume highly processed products, and their diet is softer, therefore less energy must be put into mastication, which results in lower biomechanical forces affecting the masticatory apparatus^35^. Combing the knowledge about differences in diet in medieval and modern populations with the Wolff’s law, which describes the role of mechanical stimulation of the bone tissue and how bone responds to it, we can expect, that aging processes of the facial bones were different in the past than today. Differences should be most pronounced in the maxilla and mandible because of their straightforward relationship with chewing, but we have to bear in mind, that those biomechanical processes do not occur in isolation. Forces produced during mastication also affect other areas of the facial skeleton, including orbits and *piriform* aperture^36^. Those structures are also associated with aging of the viscerocranium. Greater biomechanical loads generated by the medieval diet could have a negative impact on aging processes of the facial bones.

The aim of this study is to identify changes associated with the aging process in the facial skeleton in a medieval sample from the territory of present-day Poland and to evaluate if the nature and direction of those processes are consistent with publisheddata for modern populations. Considering that the maxilla is the bone most prone to resorption, we also aim to test the hypothesis about diet-related slower resorption of maxillary height in medieval versus modern times.

## Material

### Material

Human skeletal remains used for the analysis was recovered from an archaeological proto-urban site in Cedynia, Poland. The socio-economic features of the site have been characterized elsewhere^37^. The human remains date from 10th to fourteenth century AD. The study embraced 230 skulls of adult individuals, males and females (Table 1), however some analyses included fewer number of individuals, because of post-mortem damage (see 4. Results). Two groups of individuals were included, differing in their dental status: dentate and edentulous. In the dentate group, each individual has been assigned to one of three age categories: young adult (YA), middle adult (MA), and old adult (OA). Additional selection criteria were applied to maxilla measurements, because of its height being affected by ante mortem tooth loss. To avoid such an influence, individuals with resorption at any measurements point were excluded from the analysis. Exception to this rule was the group of old adults, where cases with single ante mortem tooth loss were taken into consideration, because of low number of senile individuals with all teeth present prior to death. For all other measurements, the individuals with no more than a single ante mortem tooth loss in maxilla and mandible qualified for the study in the dentate group.

**Table 1:** The examined sample by age, sex, and dental status.

Age category	Females		Males		Total	
	Dentate	Edentulous	Dentate	Edentulous	N	%
Young adult (YA 25–35 yrs)	46	0	45	0	91	40
Middle adult (MA 35–50 yrs)	45	9	47	12	113	49
Old adult (OA 50 + yrs)	10	0	16	0	26	11
Total	101	9	108	12	230	100

The edentulous group embraced those individuals who had lost all of their teeth during lifetime and displayed resorption of the entire alveolar process. This group was established to allow direct comparisons with living individuals, for whom the measurement data are available from the literature. As a result of low number of edentulous old adults in the osteological sample of Cedynia, this analysis was conducted for MA individuals with complete antemortem tooth loss.

## Methods

### Age and sex distribution

Sex assessment was performed based on cranial and pelvic features^38^. Age was assessed through pubic symphysis changes^39^ and dental wear^40^. As a complementary method, cranial sutures closure was used^41^.

### 3D Scanning

The skulls were scanned using a white light 3D ScanBright scanner (detector resolution up to 5 Mpix and point accuracy between 0.08 and 0.5 mm, www.smarttech3d.com) and Mesh3D software made by SMARTTECH, Poland to achieve three dimensional models of the facial skeleton. The point clouds created this way was cleaned and converted to 3D models using Geomagic Qualify 12 software (3D systems, USA).

### Measurements

**Figure 1:**
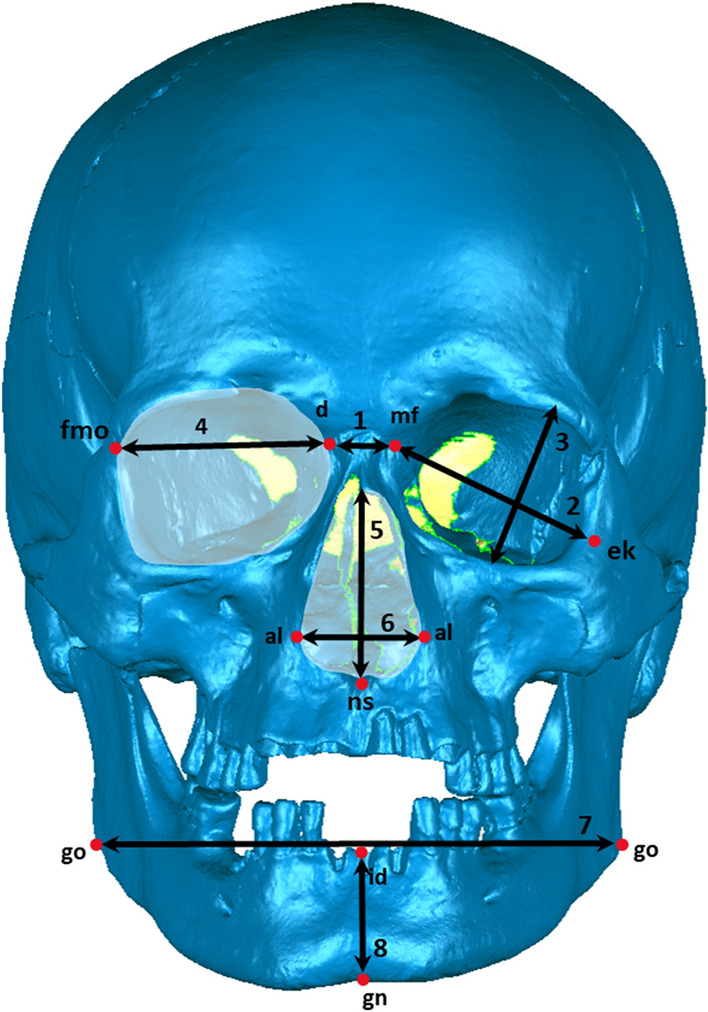
Measurements of facial skeleton: frontal view.

All measurements were taken by the first author of the study, with the use of Geomagic Qualify 12 software. Four elements of the facial skeleton: orbits, *piriform* aperture, maxilla and mandible were measured (Table 2 and Fig. 1). To maintain reproducibility of measurements while working on 3D models, the first step was to create two reference planes: the sagittal and Frankfort plane. To establish the sagittal plane, it was necessary to first determine anthropometric landmarks in the medial line of the skull (*nasion, nasospinale* and *gnathion*), and then to create the plane through connecting those points. The Frankfort plane was established through joining *porion* and two *orbitale* landmarks (Supplementary Table S1).

**Table 2:** Measurements included in the study.

Measurement	Abbreviation	Description	Figure (measurement)
Orbits			
Interorbital distance	OB-IT	Distance between left and right *maxillofrontale*^42,43^	1 (1)
Breadth	OB-B	Distance between *maxillofrontale* and *ektokonchion*^43^	1 (2)
Height	OB-H	Distance between superior and inferior orbital rim, perpendicular to the long axis of the orbit^42^	1 (3)
X-axis	OB-X	Distance from the posterior lacrimal crest to the *frontomalare orbitale* point ^26,49^	1 (4)
Surface area	OB-A	Orbital surface area	1
Distance to superior rim	OB[s]10–OB[s]90	Distance from X axis to the superior orbital rim^26,49^	2
Distance to inferior rim	OB[i]10–OB[i]10	Distance from X axis to the inferior orbital rim^26,49^	2
Distance from superior to inferior rim	OB[h]10–OB[h]90	Distance from the superior and inferior orbital rim^26,49^	2
* Piriform* aperture			
Height	PA-H	Maximal height of the *piriform* aperture	1 (5)
Breadth	PA-B	Maximal breadth of the *piriform* aperture measured as a distance between two *alare* points^38^	1 (6)
Surface area	PA-A	*Piriform* aperture surface area	1
Maxilla			
Height at the midline	MX-ML	Distance from the point marked between two first upper incisors to the or-or line measured in a straight line^69^	3
Height at the line of the P1	MX-P1	Distance from the point marked on the lowest part of the first premolar socket to the or-or line measured in a straight line^69^	3
Height at the line of the M1	MX-M1	Distance from the point marked on the lowest part of the first molar socket to the or-or line measured in a straight line^69^	3
Bigonial width	MD-W	Distance between two *gonion* points^42^	1 (7)
Chin height	MD-H	Distance from *infradentale* to *gnathion*^42^	1 (8)
Body length	MD-L	Distance from anterior most margin of the chin and the central point of the projected straight line along the posterior border of the two mandibular angles^42^	4 (1)
Maximum ramus height	MD-RH	Distance from the highest point on the condyle to the point *gonion*^42^	4 (2)
Minimum ramus width	MD-RW	Least breadth of the mandibular ramus, measured perpendicular to the height of the ramus^42^	4 (3)

Linear measurements were taken in mm and area in mm^2^.

#### Orbits

**Figure 2:**
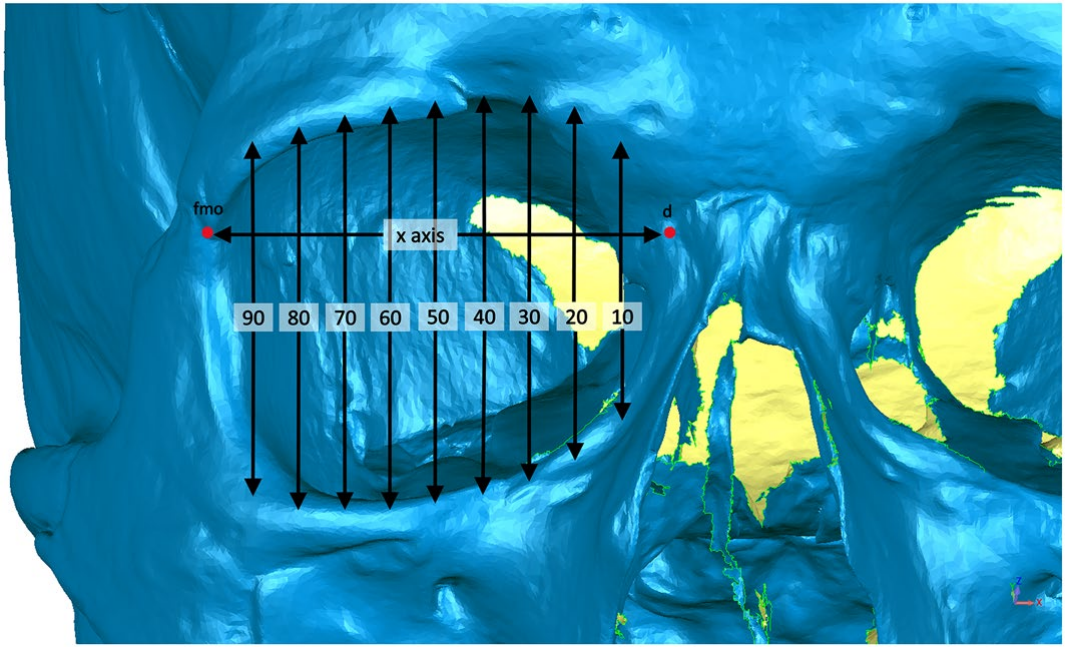
Orbital measurements: 10–90 deciles.

Orbital breadth, height, X-axis and surface area were measured (Table 2). Additionally, the measurement of interorbital length was taken. To perform the remaining measurements of the orbits, a new plane was created. It had to be parallel to the Frankfort plane and go through the frontomalare orbitale point (fmo). This plane was then used to determine the location of the point d on the surface of the posterior lacrimal crest. Connecting those two landmarks (fmo-d) established the X-axis. This axis was then divided into ten equal segments (deciles), which resulted in nine measurement points located at equal intervals along the entire length of the X-axis (referred to as deciles from 10 to 90). Through every point a plane parallel to the sagittal plane was created. They were later used to locate points on the upper and lower edge of the orbit at their intersection. Using created points on X-axis and on orbital edges OB[s]10–90, Ob[i]10–90, OB[h]10–90 measurements could be performed (Table 2 and Fig. 2).

#### *Piriform* aperture

Linear measurements of height and breadth of the *piriform* aperture was performed. Surface area of the aperture was also measured (Table 2).

#### Maxilla

**Figure 3:**
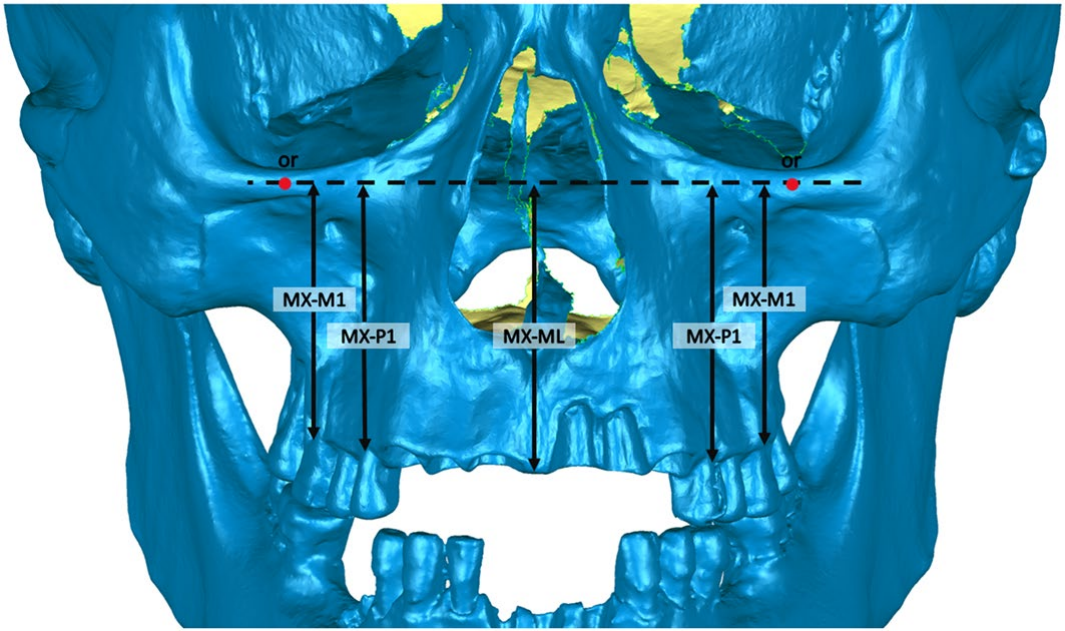
Measurements of maxillary body height.

The height of the maxillary body was measured at midline, first premolar and first molar. Two last mentioned measurements were taken bilaterally. To take all measurements, the sagittal and Frankfort planes were used as reference planes. The next step was to establish the points on the alveolar process: between the two first incisors, and in the middle of the first premolar and molar sockets. The plains perpendicular to the or-or line were drawn through those points. At the intersection of those plains with the Frankfort plane, the points were located. Together with the points on the alveolar process, they were used to perform maxillary measurements (Table 2 and Fig. 3). The choice of the maxillary measurements was dictated by the measurements performed by other authors for modern populations, available from the literature.

The availability of comparative data on edentulous and dentate individuals was used to determine the percentage reduction of the maxilla. This parameter was calculated in accordance with the formula (Eq. 1):

1 $$\frac{{{\text{HoD}} - {\text{HoE}}}}{{{\text{HoD}}}}{ } \times { }100$$

in which HoD is the height of the dentate maxilla and HoE is the height of the edentulous maxilla^44^.

The generated data were compared with publisheddata for modern populations. If authors have not calculated this parameter, it was determined based on the data included in their papers.

#### Mandible

**Figure 4:**
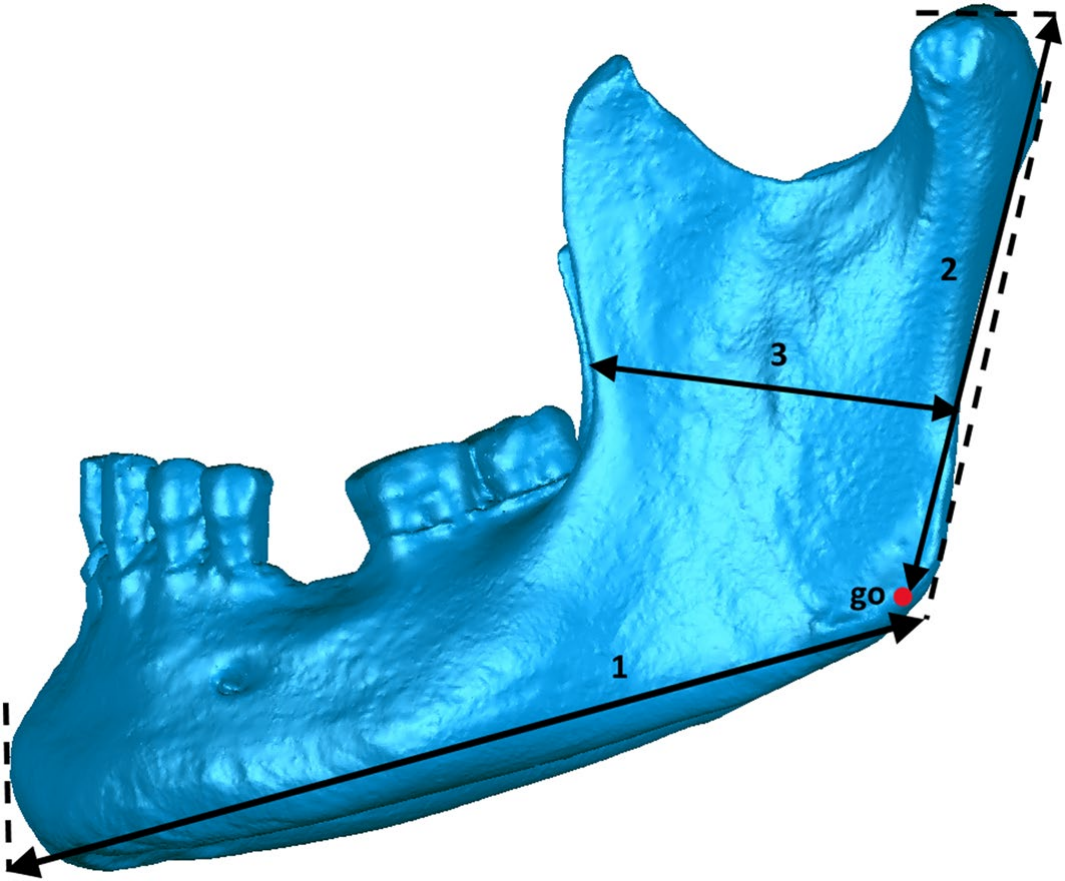
Measurements of mandible.

Chin height, bigonial breadth and body length of the mandible were measured, together with maximum height and minimum breadth of both mandibular rami (Table 2 and Fig. 4).

### Statistical analysis

Prior to performing statistical procedures, the outliers have been removed from the database to avoid their interference with the analysis. Non-parametric statistical tests were used: Kruskal–Wallis ANOVA to identify trends among age categories, and U-Mann–Whitney test to examine the differences between dentulous and edentulous individuals. To reveal potential differences between left and right side of the skull and in sex subgroups U-Mann Whitney test was performed. In order to show measurement sensitivity and specificity in sex assessment we applied ROC curves. Additionally, to detect fluctuating asymmetry in the examined skulls we calculated FA1 ([R − L|) and FA2 (|R − L|)/[(R + L)/2)] indices in accordance with the Palmer's classification^45^. Repeated measurements of vertical and horizontal parameters of orbits and *piriform* aperture were taken on 10 randomly chosen skulls by the same observer to establish the intraobserver error. Consequently, absolute differences for 6 measurements were taken into account (left and right OB-B and OB-H, PA-H, PA-B). To determine differences between repeated and initial measurements a Student's t test was applied. The statistical analyses were performed with Statistica (TIBCO Software Inc., USA), while for ROC curves SPSS Statistics (IBM, USA) was used.

## Results

For almost all measurements, differences between the male and female group in the young and middle adult age categories were statistically significant, with males reaching higher values (Table 3). ROC curves analysis revealed that the highest values of the area under the ROC curve (AUC) were obtained for mandibular parameters. ROC curves, together with AUC, are presented in Supplementary Information (Supplementary Fig. S2 and Table S3). Measurements done for orbits, maxilla and mandible showed no significant difference between the left and right side both in females and males. Descriptive data for FA1 and FA2 indices are included in Table S4 (Supplementary Table S4). Intraobserver error calculated for repeated measurements was as follows: 0.32 (left OB-B), 0.81 (right OB-B), 0.59 (left OB-H), 0.32 (right OB-H), 0.53 (PA-H), 0.22 (PA-B) and proved to be insignificant.

**Table 3:** *P*-values for differences between females and males in each age category and edentulous group.

Measurement		Side	Young adult	Middle adult	Old adult	Edentulous
Orbit	OB-IT		0.28	0.57	0.41	0.31
	OB-B	Left	0.06	0.11	0.02*	0.27
		Right	0.001*	0.15	0.02*	0.85
	OB-H	Left	0.95	0.79	0.95	0.43
		Right	0.90	0.73	0.24	0.77
	OB-X	Left	0.001*	0.01*	0.01*	0.87
		Right	0.01*	0.02*	0.07	0.80
	OB-A	Left	0.001*	0.08	0.02*	0.71
		Right	0.01*	0.38	0.03*	0.80
*Piriform* aperture	PA-H		0.28	0.03*	0.02*	0.36
	PA-B		0.02*	0.01*	0.14	0.06
	PA-A		0.07	0.01*	0.04*	0.17
Maxilla	MX-ML		< 0.001*	< 0.001*	0.11	0.01*
	MX-P1	Left	< 0.001*	< 0.001*	0.25	0.01*
		Right	< 0.001*	< 0.001*	0.13	0.02*
	MX-M1	Left	< 0.001*	< 0.001*	0.10	0.02*
		Right	< 0.001*	< 0.001*	0.06	0.11
Mandible	MD-B		< 0.001*	< 0.001*	0.04*	0.11
	MD-H		< 0.001*	< 0.001*	0.03*	0.90
	MD-L	Left	< 0.001*	< 0.001*	0.18	0.07
		Right	< 0.001*	< 0.001*	0.19	0.07
	MD-RH	Left	< 0.001*	< 0.001*	0.07	0.07
		Right	< 0.001*	< 0.001*	0.03*	0.07
	MD-RW	Left	< 0.001*	0.009*	0.01*	0.18
		Right	0.001*	0.003*	0.08	0.54

*statistically significant for *p* < 0.05, Mann–Whitney U test.

Abbreviations. Orbits: *OB-IT* Interorbital distance, *OB-B* breadth, *OB-H* height, *OB-X* X-axis, *OB-A* surface area. *Piriform* aperture: *PA-H* height, *PA-B* breadth, *PA-A* surface area. Maxilla: *MX-ML* height at the midline, *MX-P1* height at P1, *MX-MA* height at M1. Mandible: *MD-W* width, *MD-H* chin height, *MD-L* body length, *MD-RH* maximum ramus height, *MD-RW* minimum ramus width.

### Age differences

#### Orbits

In males there was a statistically significant increase in values in older age categories for right OB-H and left OB-B between YA and OA groups. The remaining measurements in males except for OB-IT showed similar, but an insignificant tendency for greater values in older age categories. In females statistically significant differences have not been found and a non-statistically significant tendency for increasing the values with age was apparent only in the case of left and right OB-X, right OB-B and left OB-B. The increase was observed for all measurements between YA and MA groups but was not continued in OA group (Table 4).

**Table 4:** Results of measurements in age groups.

Measurements			Females										Males									
			Young adult			Middle adult			Old adult			*p* value	Young adult			Middle adult			Old adult			*p* value
			N	${\overline{\text{x}}}$	SD	N	${\overline{\text{x}}}$	SD	N	x̅	SD		N	${\overline{\text{x}}}$	SD	N	${\overline{\text{x}}}$	SD	N	${\overline{\text{x}}}$	SD	
Orbit	OB-IT		28	16.47	2.65	28	17.09	2.55	10	15.90	1.70	0.39	27	17.35	1.70	28	17.49	2.41	14	16.57	1.59	0.41
	OB-B	Left	29	42.85	1.96	29	43.55	2.24	8	44.18	1.44	0.19	27	**43.97**	1.68	29	44.73	2.09	13	**46.24**	2.06	0.01*
		Right	28	42.99	1.52	29	43.69	2.05	10	44.05	1.24	0.23	29	44.23	2.11	28	44.75	2.37	12	45.83	1.87	0.24
	OB-H	Left	28	34.59	1.56	29	35.06	1.93	9	34.73	2.08	0.20	28	34.59	1.78	29	34.91	1.68	13	35.15	1.85	0.33
		Right	29	34.29	1.67	29	35.31	2.14	10	35.00	2.35	0.31	30	**34.52**	1.88	29	35.07	1.93	12	**36.00**	1.69	0.046*
	OB-X	Left	28	40.87	1.88	28	41.49	1.62	8	41.52	1.56	0.63	27	42.42	1.92	29	42.94	2.29	13	43.59	1.27	0.21
		Right	29	41.21	1.52	29	41.37	2.14	10	41.67	1.53	0.76	29	42.38	2.10	29	42.97	2.40	12	43.35	1.75	0.56
	OB-A	Left	29	1250.48	95.17	29	1303.49	90.21	8	1262.55	69.56	0.10	29	1344.46	128.74	28	1345.54	80.29	13	1350.75	70.17	0.98
		Right	29	1236.86	89.18	29	1287.44	94.31	10	1237.55	96.89	0.08	30	1303.15	95.38	29	1314.26	104.80	11	1335.63	61.92	0.19
*Piriform* aperture	PA-H		29	32.56	2.40	27	33.19	2.20	4	32.03	2.58	0.47	29	33.72	2.52	29	35.19	2.82	11	35.83	2.46	0.21
	PA-B		38	24.10	1.91	35	24.17	1.39	10	24.71	2.37	0.54	37	25.09	2.04	39	25.32	1.79	13	25.68	2.02	0.05
	PA-A		28	560.84	68.44	27	589.97	65.14	4	600.34	42.47	0.31	29	**611.08**	67.56	28	660.87	78.28	11	**701.29**	58.42	0.003*
Maxilla	MX-ML		30	36.14	2.40	30	34.72	3.02	9	34.38	3.74	0.08	30	39.01	2.46	30	38.91	3.21	11	37.83	5.30	0.87
	MX-P1	Left	30	34.47	2.31	30	32.97	2.43	9	33.39	4.92	0.07	30	37.89	2.30	30	36.96	2.93	11	36.65	5.01	0.49
		Right	30	**34.53**	2.40	29	**32.54**	3.00	9	32.19	4.00	0.03*	30	37.71	2.60	30	36.43	3.31	11	36.02	5.65	0.34
	MX-M1	Left	30	35.96	2.58	17	34.83	3.27	9	27.05	4.69	0.17	30	39.44	2.54	30	38.77	2.80	11	36.40	5.45	0.30
		Right	30	**35.73**	2.36	16	34.66	3.32	9	**31.44**	4.24	0.02*	30	39.40	2.45	30	38.28	3.13	11	36.32	6.38	0.18
Mandible	MD-B		28	94.24	5.36	28	93.59	5.41	8	94.18	4.89	0.93	29	99.80	6.09	28	102.39	4.42	8	101.45	6.67	0.17
	MD-H		30	23.66	2.99	29	23.36	2.92	7	24.79	1.68	0.84	32	27.11	2.84	30	27.69	2.90	9	27.70	2.42	0.83
	MD-L	Left	29	81.19	3.41	29	81.60	4.55	8	83.89	3.59	0.22	29	86.34	3.70	29	88.25	5.09	7	86.84	4.02	0.53
		Right	30	81.26	3.77	27	80.82	3.73	8	84.28	4.06	0.13	32	86.84	3.63	30	88.09	4.74	9	86.93	4.65	0.68
	MD-RH	Left	25	53.55	4.36	23	54.55	4.06	4	51.97	4.50	0.54	25	58.28	3.37	27	59.02	4.11	6	58.24	1.83	0.37
		Right	28	**54.24**	4.13	21	**55.23**	3.58	6	**49.77**	2.16	0.02*	29	58.97	4.02	28	60.60	3.85	7	59.30	3.42	0.43
	MD-RW	Left	29	26.79	2.54	29	26.85	2.30	7	25.12	1.85	0.13	30	29.79	2.57	30	29.11	3.05	8	29.76	2.76	0.62
		Right	29	26.89	3.10	28	26.61	2.64	8	26.23	2.09	0.74	31	29.60	2.80	29	29.25	3.36	9	29.69	3.81	0.86

*Statistically significant for *p* < 0.05, Kruskal–Wallis ANOVA. Bolded values are statistically significant differences.

Abbreviations. Orbits: *OB-IT* Interorbital distance, *OB-B* breadth, *OB-H* height, *OB-X* X-axis, *OB-A* surface area. *Piriform* aperture: *PA-H* height, *PA-B* breadth, *PA-A* surface area. Maxilla: *MX-ML* height at midline, *MX-P1* height at P1, *MX-MA* height at M1. Mandible: *MD-W* width, *MD-H* chin height, *MD-L* body length, *MD-RH* maximum ramus height, *MD-RW* minimum ramus width.

In females, both left and right orbits distance from X-axis to the superior and inferior rim increased with age between YA and MA groups in every measured point. In males this tendency was observed only for measurements of the inferior segment of both orbits, but was continued through all age groups, while in the case of OB[s]10-OB[s]90 a reverse tendency was found.

In females, a statistically significant increase was present in points corresponding to the outermost elements of the superior orbital rim (OB[s]10-OB[s]30 and OB[s]80-OB[s]90). In males, a reverse tendency was found, while the observed decrease was statistically significant in the measurements taken at the superomedial part of the orbit (OB[s]10-OB[s]40) (Table 5).

**Table 5:** Results of orbital measurements OB[s]10–90 (decile distances to superior rim) and OB[i]10–90 (decile distances to inferior rim) in age groups.

Decile	Measurement	Side	Females										Males									
			Young adult			Middle adult			Old adult			*p* value	Young adult			Middle adult			Old adult			*p* value
			N	${\overline{\text{x}}}$	SD	N	${\overline{\text{x}}}$	SD	N	${\overline{\text{x}}}$	SD		N	${\overline{\text{x}}}$	SD	N	${\overline{\text{x}}}$	SD	N	x ${\overline{\text{x}}}$	SD	
10	Upper rim	L	31	9.63	1.86	31	11.04	2.19	8	9.55	1.69	0.06	26	**11.38**	2.25	28	10.97	1.86	14	**9.70**	2.52	0.01*
		R	31	**8.88**	2.14	30	**10.23**	1.81	10	8.41	1.84	0.02*	25	**10.34**	1.72	29	10.45	2.05	14	**8.69**	1.86	0.01*
	Lower rim	L	30	17.50	2.27	30	17.88	2.75	8	16.59	2.05	0.37	26	17.84	1.98	28	17.70	2.14	14	17.16	2.62	0.44
		R	31	17.09	2.11	31	17.48	2.67	10	16.09	2.25	0.39	25	17.77	1.99	27	17.67	1.98	14	17.66	2.24	0.89
20	Upper rim	L	31	**11.79**	1.84	31	**13.23**	1.97	8	11.73	1.76	0.02*	26	**13.61**	2.26	28	13.24	1.49	14	**12.27**	2.22	0.02*
		R	31	11.45	2.11	31	12.45	2.00	10	10.88	1.97	0.11	25	12.68	1.75	27	12.53	1.38	14	11.22	1.48	0.05
	Lower rim	L	30	20.41	2.13	31	20.75	2.48	8	19.65	1.97	0.38	26	20.78	1.60	28	20.85	1.89	14	20.36	2.36	0.54
		R	31	20.47	1.97	31	20.41	2.56	10	19.37	2.06	0.32	25	20.74	1.68	27	20.93	1.74	14	21.16	2.10	0.75
30	Upper rim	L	31	**12.74**	1.77	31	**13.94**	1.86	8	12.33	1.76	0.02*	26	**14.62**	2.15	28	14.06	1.57	14	**13.20**	1.70	0.02*
		R	31	12.74	1.97	31	13.61	1.95	10	12.76	2.07	0.23	25	14.14	1.87	28	13.90	1.26	14	13.01	1.59	0.05
	Lower rim	L	30	22.86	1.96	31	22.98	2.22	8	22.29	1.99	0.61	26	22.87	1.47	28	23.25	1.68	14	23.09	2.27	0.47
		R	31	22.74	1.73	31	22.93	2.36	10	22.03	2.15	0.65	25	23.13	1.52	27	23.40	1.83	14	23.77	2.03	0.50
40	Upper rim	L	31	12.97	1.63	31	13.89	1.68	8	12.54	1.90	0.06	26	**14.43**	1.75	28	13.86	1.37	14	**13.25**	1.67	0.03*
		R	31	13.04	1.69	31	13.85	1.94	10	13.29	2.39	0.28	25	**14.47**	1.51	28	13.96	1.23	14	**13.29**	1.39	0.02*
	Lower rim	L	30	24.55	1.80	31	24.55	2.01	8	24.33	2.08	0.84	26	24.49	1.69	28	24.94	1.94	14	24.94	2.06	0.31
		R	31	24.48	1.53	31	24.71	2.29	10	23.91	1.89	0.53	25	24.81	1.50	27	24.95	1.77	14	25.74	1.96	0.21
50	Upper rim	L	31	12.61	1.50	31	13.39	1.51	8	12.56	1.70	0.15	26	13.94	1.64	28	13.46	1.25	14	13.05	1.59	0.07
		R	31	12.79	1.42	31	13.61	1.88	10	13.21	2.24	0.30	25	14.07	1.39	28	13.59	1.21	14	13.41	1.43	0.10
	Lower rim	L	30	25.62	1.56	31	25.72	1.81	8	25.96	1.80	0.96	26	26.04	2.59	28	26.08	1.87	14	26.21	1.85	0.49
		R	31	25.53	1.51	31	25.80	2.23	10	25.29	1.76	0.59	25	25.98	1.53	27	26.02	1.72	14	26.92	1.81	0.13
60	Upper rim	L	31	12.09	1.35	31	12.85	1.33	8	12.31	1.55	0.09	26	13.16	1.53	28	12.87	1.15	14	12.53	1.48	0.25
		R	31	12.27	1.29	31	12.97	1.79	10	12.84	2.21	0.33	25	13.35	1.36	28	13.14	1.17	14	13.09	1.50	0.31
	Lower rim	L	30	26.20	1.46	31	26.43	1.82	8	26.82	1.84	0.63	26	26.31	1.62	28	26.68	1.79	14	26.85	1.80	0.45
		R	31	25.99	1.51	31	26.38	2.18	10	26.02	1.58	0.52	25	26.51	1.55	27	26.58	1.71	14	27.44	1.85	0.16
70	Upper rim	L	31	11.14	1.20	31	11.86	1.34	8	11.33	1.55	0.12	26	11.98	1.49	28	11.79	1.11	14	11.63	1.30	0.53
		R	31	11.33	1.17	31	11.91	1.68	10	12.01	2.19	0.34	25	12.30	1.25	27	12.23	1.16	14	12.23	1.47	0.49
	Lower rim	L	30	26.34	1.48	31	26.56	1.87	8	27.09	1.81	0.43	26	26.49	1.78	28	26.76	1.72	14	27.01	1.86	0.54
		R	31	26.12	1.57	31	26.56	2.21	10	26.29	1.60	0.54	25	26.63	1.50	27	26.57	1.73	14	27.54	1.98	0.21
80	Upper rim	L	31	**9.50**	1.12	31	**10.33**	1.37	8	9.86	1.49	0.046*	26	10.41	1.44	28	10.13	1.21	14	10.09	1.43	0.65
		R	31	9.70	1.18	31	10.37	1.59	10	10.48	2.08	0.04*	25	10.74	1.22	27	10.70	1.19	14	10.67	1.42	0.68
	Lower rim	L	30	25.90	1.50	31	26.05	2.05	8	26.73	1.81	0.55	26	26.01	1.83	28	26.26	1.76	14	26.64	2.19	0.49
		R	31	25.74	1.56	31	26.05	2.25	10	25.79	1.50	0.71	25	26.19	1.59	27	26.06	1.76	14	27.06	2.25	0.33
90	Upper rim	L	31	**6.58**	0.98	31	**7.61**	1.29	8	7.25	1.23	0.045*	26	7.75	1.24	28	7.40	1.26	14	7.53	1.46	0.54
		R	31	6.94	1.39	31	**7.87**	1.52	10	**7.92**	1.61	0.04*	25	8.06	1.24	27	8.20	1.30	14	8.12	1.57	0.92
	Lower rim	L	30	24.13	1.75	31	23.93	2.64	8	25.07	1.85	0.38	26	23.94	2.15	28	24.15	1.91	14	25.11	2.64	0.32
		R	31	23.49	2.32	31	23.44	2.67	10	23.05	1.75	0.64	25	23.75	2.14	27	23.73	2.08	14	24.46	2.75	0.62

*Statistically significant for *p* < 0.05, Mann–Whitney U Test. Bolded values are statistically significant differences.

Abbreviations: *L* left, *R* right.

Height at particular deciles showed a slight statistically significant increase in value for right OB[h]10 (YA vs. OA) and both sides in OB[h]20 for females (left-YA vs. MA, right-YA vs. OA) (Supplementary Table S5).

#### *Piriform* aperture

All measurements had higher values in older age groups for both sexes, except for PA-H in females, but the differences were statistically significant only in males in the case of PA-A (YA vs. OA) (Table 4).

#### Maxilla

All maxillary parameters decreased with age, besides left MX-P1 in females. Statistically significant differences were noticed only for females for right MX-P1 (YA vs. MA) and right MX-M1 (YA vs. OA) (Table 4).

#### Mandible

In both female and male groups, no changes in mandibular dimensions with age were noticed (Table 4). Mean values differed only slightly between age groups, except in females, where MD-RH significantly increased between YA and MA groups and decreased in OA group.

### Differences between dentate and edentulous middle adults

For almost all maxillary measurements, differences between males and females were statistically significant in the edentulous group. Measurements for orbits, maxilla and mandible did not show a significant difference between the left and right side both in females and males.

#### Orbits

The measurements of the orbits tend to reach higher values in edentulous individuals. Most of the measured parameters show this pattern, except for left and right OB-A, and left OB-H in both sexes. Differences between dentate and edentulous individuals were statistically significant for OB-IT, left OB-B, right and left OB-X for females and OB-IT, right and left OB-A, and left OB-B for males (Table 6). Edentulous individuals tend to have lower values of the distance from X-axis to the superior and inferior orbital rim. Only in the case of the lateral part of the upper orbital ridge (60–90 deciles), higher values of this distance are present in both sex groups. In females, the results are statistically significant in both left and right OB[s]10-OB[s]20, and in males in the left OB[s]10-OB[s]30. For both groups statistically significant differences in distances to the inferior rim were noted in points corresponding to the medial part of the inferior orbital rim (OB[i]10-OB[i]30) (Table 7). A decrease in orbital height measurements for particular deciles in females was statistically significant for the superior part of the orbit (OB[h]10-Ob[h]30 for both sides). Measurements of the medial part of the orbital rims (OB[h]10–40 for left side and OB[h]10-Ob[h]20 for right) were statistically significant in males (Supplementary Table S6).

**Table 6:** Results of measurements in dentate and edentulous individuals.

Measurements			Female							Male						
			Dentate			Edentulous			*p* value	Dentate			Edentulous			*p* value
			N	${\overline{\text{x}}}$	SD	N	${\overline{\text{x}}}$	SD		N	${\overline{\text{x}}}$	SD	N	${\overline{\text{x}}}$	SD	
Orbit	OB-IT		28	**17.09**	2..55	7	**14.58**	1.23	0.01*	28	**17.49**	2.41	7	**13.89**	1.24	0.01*
	OB-B	Left	29	**43.55**	2.24	8	**45.58**	2.09	0.03*	29	**44.73**	2.09	8	**46.54**	1.86	0.04*
		Right	28	43.69	2.05	7	45.69	2.59	0.048*	28	44.75	2.37	7	46.03	1.82	0.17
	OB-H	Left	29	35.06	1.93	8	35.49	2.16	0.68	29	34.91	1.68	8	34.96	1.90	0.80
		Right	29	35.31	2.14	8	35.25	131.48	0.96	29	35.07	1.93	7	34.89	1.87	0.65
	OB-X	Left	29	**41.49**	1.62	8	**43.96**	1.87	0.004*	29	42.94	2.29	8	44.20	1.46	0.14
		Right	29	**41.37**	2.14	7	**43.53**	1.74	0.03*	29	42.97	2.40	7	43.90	1.29	0.18
	OB-A	Left	28	1303.49	90.21	8	1262.48	2.15	0.24	28	**1345.54**	80.29	8	**1251.14**	77.19	0.01*
		Right	29	1287.44	94.31	7	1217.39	80.86	0.14	29	**1314.26**	104.80	7	**1226.63**	77.57	0.04*
*Piriform* aperture	PA-H		27	33.19	2.20	5	32.73	2.24	0.76	29	35.19	2.82	11	34.29	2.46	0.45
	PA-B		35	24.17	1.39	8	23.85	1.79	0.68	39	25.32	1.79	11	25.76	2.02	0.56
	PA-A		27	589.97	65.14	5	546.18	92.04	0.12	28	660.87	78.28	11	625.25	79.10	0.39
Maxilla	MX-ML		30	**34.72**	3.02	9	**29.73**	4.62	0.006*	30	**38.91**	3.21	9	**35.89**	2.58	0.02*
	MX-P1	Left	30	**32.97**	2.43	8	**28.04**	3.30	< 0.001*	30	**36.96**	2.93	9	**32.37**	1.80	0.004*
		Right	29	**32.54**	3	8	**27.69**	2.69	0.001*	30	**36.43**	3.31	9	**31.83**	3.10	0.002*
	MX-M1	Left	17	**34.83**	3.27	8	**29.17**	3.52	< 0.001*	30	**38.77**	2.80	9	**33.71**	2.01	< 0.001*
		Right	16	**34.66**	3.32	8	**29.72**	2.51	0.002*	30	**38.28**	3.13	9	**32.86**	4.36	< 0.001*
Mandible	MD-B		28	93.59	5.41	5	93.92	6.76	0.90	28	102.39	4.42	4	101.6	5.37	0.80
	MD-H	Right	28	23.66	2.99	4	20.20	3.10	0.19	30	**27.69**	2.90	4	**20.27**	7.44	0.01*
	MD-L	Left	29	81.60	4.55	5	79.78	5.20	0.70	29	88.25	5.09	4	87.30	3.52	0.81
		Right	27	80.82	3.73	5	80.17	4.95	0.98	30	88.09	4.74	4	88.83	4.65	0.57
	MD-RH	Left	23	**54.55**	4.06	5	**48.78**	4.79	0.02*	27	59.02	4.11	3	56.55	4.00	0.41
		Right	21	**55.23**	3.58	4	**50.80**	3.20	0.046*	28	60.60	3.85	4	58.56	3.80	0.44
	MD-RW	Left	29	26.85	2.30	5	26.75	2.01	0.91	30	29.11	3.05	4	29.41	3.33	0.91
		Right	27	80.82	3.73	5	80.17	4.95	0.98	30	88.09	4.74	4	88.83	4.65	0.57

*Statistically significant for *p* < 0.05, Kruskal–Wallis ANOVA. Bolded values are statistically significant differences.

Abbreviations. Orbits: *OB-IT* Interorbital distance, *OB-B* breadth, *OB-H* height, *OB-X* X-axis, *OB-A* surface area. *Piriform* aperture: *PA-H* height, *PA-B* breadth, *PA-A* surface area. Maxilla: *MX-ML* height at midline, *MX-P1* height at P1, *MX-MA* height at M1. Mandible: *MD-W* width, *MD-H* chin height, *MD-L* body length, *MD-RH* maximum ramus height, *MD-RW* minimum ramus width.

**Table 7:** Results of orbital measurements OB[s]10–90 (decile distances to superior rim) and OB[i]10–90 (decile distances to inferior rim) in dentate and edentulous individuals.

Decile	Measurement	Side	Females							Males						
			Dentate			Edentulous			*p* value	Dentate			Edentulous			*p* value
			N	${\overline{\text{x}}}$	SD	N	${\overline{\text{x}}}$	SD		N	${\overline{\text{x}}}$	SD	N	${\overline{\text{x}}}$	SD	
10	Upper rim	L	31	**11.04**	2.19	8	**8.38**	1.88	0.004*	28	**10.97**	1.86	9	**8.71**	2.02	0.01*
		R	30	**10.23**	1.81	7	**7.66**	1.42	0.003*	29	10.45	2.05	8	8.96	1.97	0.11
	Lower rim	L	30	**17.88**	2.75	8	**13.28**	2.53	0.001*	28	**17.70**	2.14	9	**14.33**	2.82	0.001*
		R	31	**17.48**	2.67	7	**14.38**	1.74	0.003*	27	**17.67**	1.98	8	**13.57**	2.77	0.001*
20	Upper rim	L	31	**13.23**	1.97	8	**11.06**	1.73	0.01*	28	**13.24**	1.49	9	**11.13**	2.03	0.01*
		R	31	**12.45**	2.00	7	**10.36**	1.31	0.01*	27	12.53	1.38	8	11.55	1.98	0.17
	Lower rim	L	31	**20.75**	2.48	8	**17.53**	2.11	0.001*	28	**20.85**	1.89	9	**18.62**	1.97	0.01*
		R	31	**20.41**	2.56	7	**17.93**	1.72	0.001*	27	**20.93**	1.74	8	**18.08**	1.57	0.002*
30	Upper rim	L	31	13.94	1.86	8	12.59	1.64	0.06	28	**14.06**	1.57	9	**12.73**	1.89	0.02*
		R	31	13.61	1.95	7	12.52	1.05	0.15	28	13.90	1.26	8	13.11	2.14	0.30
	Lower rim	L	31	**22.98**	2.22	8	**20.48**	2.10	0.02*	28	**23.25**	1.68	9	**21.53**	1.71	0.01*
		R	31	22.93	2.36	7	21.16	1.63	0.08	27	**23.40**	1.83	8	**20.91**	1.37	0.004*
40	Upper rim	L	31	13.89	1.68	8	13.21	1.43	0.25	28	13.86	1.37	9	13.12	2.02	0.10
		R	31	13.85	1.94	7	13.09	0.58	0.29	28	13.96	1.23	8	13.33	2.25	0.47
	Lower rim	L	31	24.55	2.01	8	22.88	1.87	0.14	28	**24.94**	1.94	9	**23.61**	1.47	0.03*
		R	31	24.71	2.29	7	23.38	1.59	0.11	27	**24.95**	1.77	8	**23.07**	1.26	0.01*
50	Upper rim	L	31	13.39	1.51	8	13.27	1.09	0.79	28	13.46	1.25	9	12.85	2.06	0.20
		R	31	13.61	1.88	7	13.21	0.48	0.64	28	13.59	1.21	8	13.31	2.18	0.52
	Lower rim	L	31	25.72	1.81	8	24.63	1.99	0.24	28	26.08	1.87	9	24.88	1.50	0.05
		R	31	25.80	2.23	7	24.88	1.68	0.31	27	**26.02**	1.72	8	**24.40**	1.18	0.03*
60	Upper rim	L	31	12.85	1.33	8	12.94	0.94	0.73	28	12.87	1.15	9	12.48	1.76	0.27
		R	31	12.97	1.79	7	12.84	0.73	0.76	28	13.14	1.17	8	13.10	1.94	0.88
	Lower rim	L	31	26.43	1.82	8	25.63	1.59	0.27	28	26.68	1.79	9	25.74	1.68	0.21
		R	31	26.38	2.18	7	25.42	1.75	0.43	27	26.58	1.71	8	25.28	1.25	0.06
70	Upper rim	L	31	11.86	1.34	8	12.02	1.00	0.86	28	11.79	1.11	9	11.74	1.50	0.51
		R	31	11.91	1.68	7	12.15	0.76	0.82	27	12.23	1.16	8	12.37	1.70	0.95
	Lower rim	L	31	26.56	1.87	8	25.82	1.45	0.30	28	26.76	1.72	9	25.96	1.82	0.23
		R	31	26.56	2.21	7	25.65	2.00	0.43	27	26.57	1.73	8	25.03	1.46	0.13
80	Upper rim	L	31	10.33	1.37	8	10.72	0.95	0.50	28	10.13	1.21	9	10.49	1.33	0.41
		R	31	10.37	1.59	7	10.93	0.76	0.30	27	10.70	1.19	8	10.98	1.35	0.60
	Lower rim	L	31	26.05	2.05	8	26.58	3.72	0.77	28	26.26	1.76	9	25.59	1.76	0.44
		R	31	26.05	2.25	7	25.26	2.03	0.42	27	26.06	1.76	8	25.03	1.46	0.19
90	Upper rim	L	31	7.61	1.29	8	8.26	0.97	0.16	28	7.40	1.26	9	7.91	1.24	0.18
		R	31	7.87	1.52	7	8.51	0.61	0.26	27	8.20	1.30	8	8.57	1.01	0.49
	Lower rim	L	31	23.93	2.64	8	22.87	1.33	0.19	28	24.15	1.91	9	23.37	2.72	0.45
		R	31	23.44	2.67	7	22.89	1.78	0.41	27	23.73	2.08	8	22.34	1.78	0.10

*Statistically significant for *p* < 0.05, Mann–Whitney U Test. Bolded values are statistically significant differences.

Abbreviations: *L* left, *R* right.

#### *Piriform* aperture

All measurements in both female and male group were lower in edentulous group except for PA-B in males but statistically insignificant (Table 6).

#### Maxilla

In males and females, a statistically significant decrease of all values between dentate and edentulous groups was noted (Table 6). In accordance with the formula described in the Material and Methods section, the percentage reduction of maxilla was calculated. For females, the values were 14.37% for midline, 14.96% for P1 and 15.28% for M1, and in males they were 7.76%, 12.53% and 13.58%, respectively.

#### Mandible

The majority of the mandibular measurements tended to decrease in edentulous group. It was statistically significant in females for right and left MD-RH and in males for MD-H (Table 6).

## Discussion

Our research revealed the character and direction of the facial skeleton aging processes in the medieval sample and compared the metric data between the medieval sample and modern populations. To our knowledge, this is the first study which has examined facial skeleton changes associated with age in a past population. We also investigated alterations in the facial bones caused by the reduction of biomechanical forces during chewing, as represented by edentulism. The most important finding confirms that the total antemortem tooth loss influences the whole craniofacial complex, even in the orbital region, which has been very often overlooked. Our study is also one of a few analyses carried out entirely in three dimensions. Even though the obtained results give a consistent image of age-related changes, they must be treated with caution, due to the main limitations of our study—cross-sectional character and limited sample sizes (see “Limitations” section). A potential confounding factor of random environmental effects, which may manifest itself as fluctuating asymmetry^46^, has been considered here. However, the performed analysis of two FA indicators showed that this variable was negligible in our sample.

### Craniofacial skeletal changes with age

#### Orbits

Left OB-B and right OB-H measurements in males showed a significant increase with age. Those results indicate that the orbits of medieval individuals increased in vertical and horizontal dimensions with age, which was caused by bone resorption of their rims, leading to an increase in orbital surface area. Those results are consistent with the data obtained for modern populations^28,47–52^. Although it should be noted that some authors failed to show significant changes with age in orbital parameters^29,53^ or even suggested a decrease in some orbital dimensions^27,54,55^. According to Karunanayake^27^ those contradictions arise from varying location of the measurements among researchers, which can be an important factor considering that resorption in orbital area is not homogeneous. In our study of the medieval population, values tended to increase with age for the medial (OB[s]10–30) and lateral (OB[s]80–90) parts of the upper rim. In males, upper edge measurements have an opposite trend, which was significant in its medial part (OB[s]10–40). For modern populations, it has been established that resorption in orbits occurs along their whole border, but superomedial and inferolateral parts manifest the greatest intensification of changes^27,29,49^. Our results suggest that upper rim resorption focused on two extreme areas (medial and distal). These changes in the medial part of the superior rim are consistent with living populations. However, the observed resorption in the lateral area of this edge is difficult to explain, because it has not been found in modern populations and this is the only element of the orbit in the immediate vicinity of the chewing muscles^56^. Decreased values in males suggests that changes in the area of the superior rim differ depending on sex. The lower edge changes had the same direction for both sexes, but an intensification of resorption in the lateral part of this edge found in living populations^27,29,49^ was not present in the examined sample at a statistically significant level. It is possible that those changes could not be demonstrated, because they manifest themselves late in life^8^, and the examined individuals were too young for this phenomenon to occur. However, it could be also attributed to stronger biomechanical forces associated with a medieval diet.

#### *Piriform* aperture

Our results suggest that *piriform* aperture edges are being resorbed with age, which causes an increase in its horizontal and vertical parameters. It is consistent with the results of other studies, which showed significant changes of this kind^26,27,47,52,54,57,58^. In our medieval sample, the only statistically significant result was the increase of the surface area in successive age groups. This suggests that changes with age in the medieval adults were less intense than in the present times, but the direction and character of changes are the same.

#### Maxilla

Maxilla undergoes resorption with age, accompanied by reduction of its vertical height. In our sample maxillary height tended to decrease, but observed differences were statistically significant only for right P1 and M1 measurements in females. The one-side significance of the results could suggest that the resorption was more prominent on the right side of the examined individuals because of the left side chewing preferences, and therefore higher biomechanical forces affecting this side of the maxilla, leading to reduction of the resorption rate. However, left and right side measurements in each age category showed no differences, so it cannot be confirmed. For modern populations, some authors reported that midfacial height increases with age^59–61^. Others showed that aging changes result in a reduction of maxillary parameters^24,27,55^. However, those studies differ in applied methodology from our study, and thus they cannot be directly compared.

#### Mandible

In the medieval population, the only significant result is the decreasing right ramus height in females. In modern individuals, the same tendency was observed by Shaw et al.^62^ and Parr et al.^63^, and the opposite by Garib^64^. Patterns of changes with age in the mandibular region are still unclear. Despite the similarities of taking measurements, the results are inconsistent. It is still debated if the mandible of elderly individuals is shaped only by tooth loss^30^ or if aging is a co-factor acting together with it^62,63^. Studies, which actually found the age effect on mandible, indicated different mandibular elements to be affected and different direction of aging processes (increase vs decrease of measurements’ values). Recent studies suggest that contradictory findings may result from varying methodology. Masticatory muscle strength was shown to correlate with the mandibular shape analyzed by geometric morphometry, and not by linear measurements^64^. Mendes^65^ proved that changes in shape occur with age and showed that males and females express different patterns of those changes. In females, they begin earlier and beside resorption in the vertical dimension in the anterior and inferior region, which similarly occurs in males, they are also present in the sagittal and lateral dimension.

#### Skull areas most susceptible to aging

**Figure 5:**
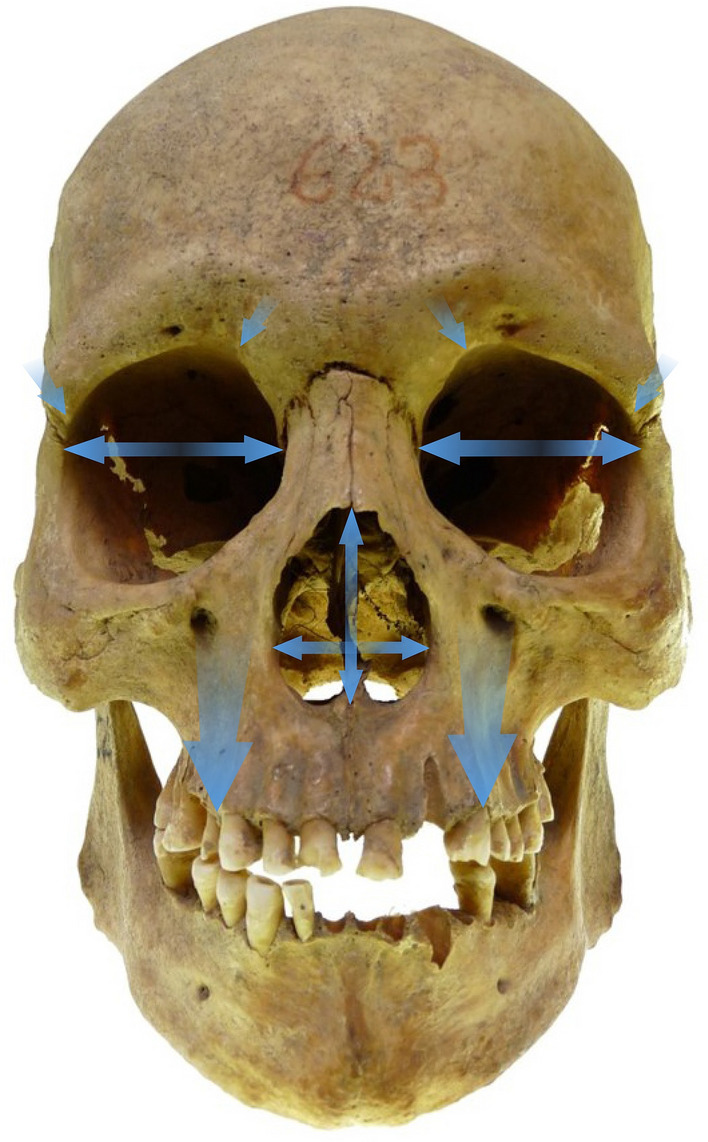
Intensification of aging changes in facial skeleton (the more intense the changes, the thicker the arrow).

The obtained results clearly confirm that with age come multiple changes in the craniofacial area, mainly consisting in specific site resorption, which is most intense in the orbits, *piriform* aperture and maxilla. With age, the margins of both the orbits and the *piriform* aperture resorb, which consequently leads to an increase in their surface area, and maxillary vertical height decrease. These changes contribute to the morphology of aging face, which is described by Mendelson and Wong^8^ as an increase of the orbital volume and surface causing deeper setting of the eyeballs in the elderly and drooping of the upper eyelids. Resorption in area of the *piriform* aperture and retraction of its lower part is responsible for weakening of the skeletal support for the soft tissues, which makes the nose appear longer. Bone loss is most intense in maxilla, because of mutual relationships of the age-related resorption and tooth loss. Intensively shrinking maxilla can significantly contribute to the nasolabial folds appearing, tear valleys (furrows located below the eye), or zygomatic eminence (a bulge in the form of a bag under the eye)^6,8,62^. The pattern of changes of the craniofacial skeleton with age as revealed in our medieval sample is presented in Fig. 5.

### Differences between dentate and edentulous individuals

#### Orbits

An increase in orbital parameters between dentate and edentulous individuals is marked mostly in the horizontal dimensions (OB-X and OB-B). It leads to reduction of interorbital distance in edentulous skulls in comparison to dentate group. A tendency for lower values in the edentulous group is present for superomedial (OB[s]10–30) and inferomedial and central (OB[i]10–50) parts of the rim. Until now, not much is known about the influence of edentulism on orbital region. Williams and Slice^66^ performed shape analysis in a modern population, which showed an association between edentulism and positioning of orbital rims: superior rim is orientated more posteriorly, and inferior rim more anteriorly, while the lateral orbital border moves superiorly and medial border inferiorly in edentulous skulls. It is possible that those changes have impacted the measured distance in the medieval sample.

#### *Piriform* aperture

A comparison of dimensions of the piriform aperture in dentate and edentulous individuals showed nonsignificant differences. This region of the facial skeleton is subject to biomechanical influences to a small extent, which has been already suggested by confirming its stability against influence of extrinsic and intrinsic factors that cause alterations of the craniofacial skeleton^20,66,67^.

#### Maxilla

**Figure 6:**
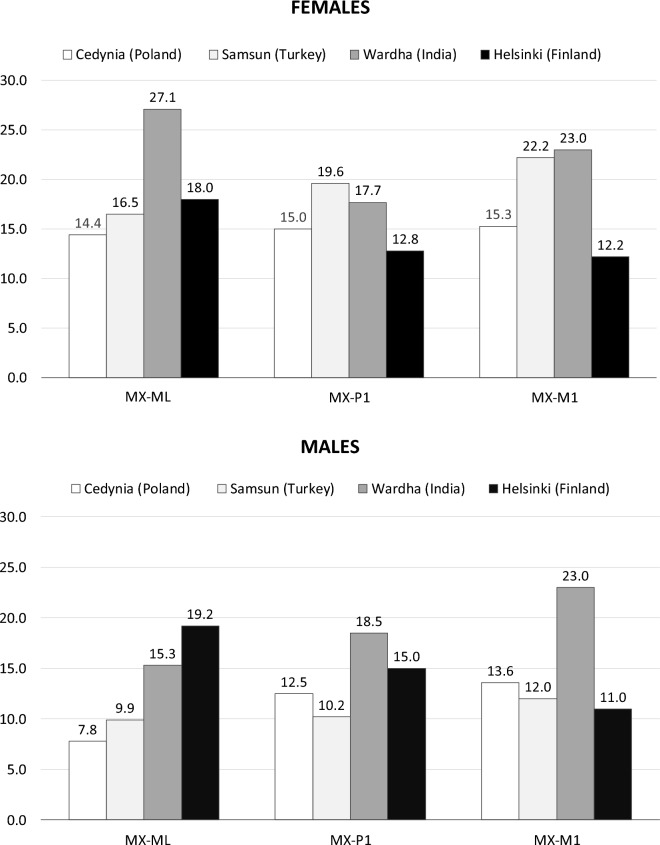
Comparison of maxillary reduction rate in medieval population from Cedynia with results of different authors for modern populations: Samsun (Turkey) – (Canger and Celenk^68^), Wardha (India) – (Panchbhai^44^), Helsinki (Finland) – (Xie et al.^69^).

In this study, all measurements of maxilla are statistically significant, which is consistent with analyzes performed in modern populations^44,68,69^. Maxillary reduction rate (see “Maxilla” section) varies from study to study. The discrepancies are most likely due to different criteria qualifying individuals to the edentulous group. Only Cangers and Celenk’s^68^ study distinguished between prosthesis users and non-users, which may be an important factor in alveolar process reduction^70^. Generally, the rate of maxillary reduction in examined medieval females and males (Fig. 6) has lower values in comparison with the living population. In the medieval population, bone loss is severe in area of posterior teeth, which is compatible with the results for modern populations^44,69^.

#### Mandible

A comparison of dentate and edentulous individuals in the examined population revealed a significant chin and ramus height reduction. Multiple studies have confirmed the relationship between tooth loss and the reduction of vertical parameters of the mandible^30,44,63,68,69,71^. Loss of teeth also causes shortening of the ramus height, which follows the widening of the gonial angle^72^. The proposed explanation for this phenomenon is a disequilibrium between the elevator and depressor muscles, with the dominant role of the latter or by the absence of the molar buttress^73^. However, it should be noted that other authors reported no correlation between tooth loss and mandibular ramus height^30,63,73^ or even observed its increase^59^.

#### Skull areas most affected by edentulism

**Figure 7:**
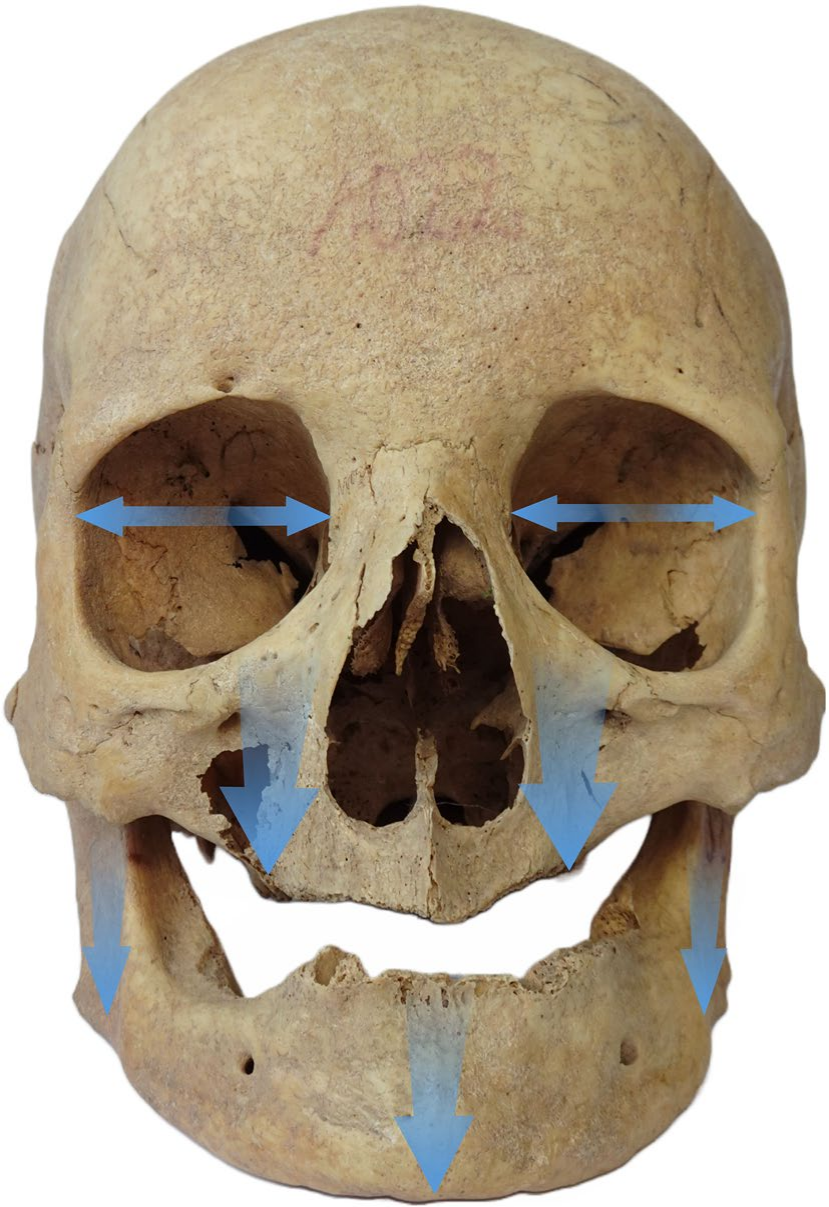
Intensification of changes caused by edentulism in facial skeleton (the more intense the changes, the thicker the arrow).

The results of comparisons between dentate and edentulous individuals in this research are mainly consistent with previous studies. Most authors have focused on changes in masticatory apparatus because of its direct relationship with tooth loss, while Williams and Slicer^66^ performed a shape analysis of influence of edentulism on the facial skeleton. They showed that the weakening of biomechanical forces produced during the chewing process affects the whole facial skeleton. This has been supported by the present study (Fig. 7). Our results indicate that the changes associated with tooth loss are less severe in the medieval population from Cedynia compared to modern populations, most likely because the observed resorption processes are susceptible to the influence of biomechanical forces, and greater biomechanical loads generated by coarser diet reduced the resorption rate.

### Limitations

The presented research is not without limitations. The most important concerns relate to the cross-sectional character of the research, which is inherent to skeletal samples. However, the examined osteological series is biologically and culturally homogeneous, as revealed by anthropological and archaeological data, and in the analyzed time period no significant socio-cultural and/or environmental changes occurred^74,75^, which, we believe, justifies our research. Additionally, it should be borne in mind, that comparative data of facial skeleton aging are scarce. Thus, contrasting our findings with modern data should be treated with caution, because of different ethnical background of the populations, which constituted the comparative database. As already suggested, facial aging processes may differ depending on the origin of the examined individuals^76^. Even the results obtained for similar ethnic groups by different authors are inconsistent.

Another difficulty relates to the small number of the oldest individuals in bioarchaeological samples, as ours. Studies of living populations include individuals over 80 years of age, which is almost impossible to acquire for past populations, due to relatively short lifespan and the difficulty in estimating age-at-death of individuals over 70 years of age, based on changes in bones and teeth. This results in limited comparability of age ranges applied in past and modern samples. Small number of old individuals can be a reason why some tendencies of changes were marked only between YA and MA groups and were not continued in OA groups. This also resulted in failure to show expected sex differences, while, generally, resorption is more intense in females, due to their hormone predispositions to bone loss^77^. In turn, scarcity of edentulous individuals made it difficult to indicate changes caused by reduction of biomechanical forces. Also, it was hard to obtain a satisfactory number of maxillary measurements at first molars, because most of them were lost during lifetime. This may result from poor dental status of females in past populations, partly caused by pregnancy and lactation^78,79^. During pregnancy the level of sex hormones increases, which is a factor predisposing to inflammatory changes in gums, and gingivitis in turn predisposes to tooth loss^80,81^.

## Conclusions

Our research revealed that the character and direction of age-related changes in the facial skeleton are similar in the examined medieval sample and modern populations. The major change is associated with maxillary height decrease, which is accompanied by enlargement of the *piriform* aperture and orbits. Our findings, which are inconsistent with previous studies, relate to the specific areas of progressive resorption and direction of changes in orbits. The rate of craniofacial complex changes with age and their intensity is lower in the examined medieval sample in comparison with modern populations, and, as such, our results correspond with the theory of the role of mechanotransduction in the facial skeleton aging process. Our research also suggests that edentulism mainly leads to reduction of the maxillary and mandibular vertical dimension, together with widening of the orbits and shortening of the mandibular ramus in toothless individuals. It should be emphasized, however, that our results are preliminary and might be affected by limited sample sizes, thus it would be advisable to carry out similar analyzes on more numerous populations with greater number of old and edentulous individuals. To obtain reliable results such research should definitely involve 3D measurements, used in our study. Moreover, considering the complexity of changes in the facial skeleton, advanced 3D shape analysis would be particularly informative. Because of contradictory findings about aging of the facial skeleton, it is recommended that a rigorous methodical approach and unified measurement protocols must be adopted.

### Supplementary Information

Supplementary Information.

## Data Availability

The data that support the findings of this study are available from the corresponding authors upon reasonable request.
