# Supplementary material for: Insight into age-related changes of the human facial skeleton based on medieval European osteological collection
Source: Sci Rep. 2023 Nov 23;13:20564. doi: 10.1038/s41598-023-47776-4 (PMC10667279; doi:10.1038/s41598-023-47776-4)
Supplement: Supplementary file 1 — Supplementary Information. [file 41598_2023_47776_MOESM1_ESM.pdf]

“Insight into age-related changes of the human facial skeleton based on medieval European osteological collection”

Anna Walczak, Marta Krenz-Niedbała, Sylwia Łukasik

Supporting Information

This document contains the following supporting information, supplementing the discussion and results presented in the article “Insight into age-related changes of the human facial skeleton based on medieval European osteological collection”:

- Supplementary Table S1 – Anthropometric points used in the study
- Supplementary Figure S2 – ROC curves for applied facial skeleton measurements.
- Supplementary Table S3 – Area under the ROC curves for applied measurements
- Supplementary Table S4 – Descriptive data of FA1 and FA2 indices for bilateral measurements.
- Supplementary Table S5 – Distance from superior to inferior orbital rim for females and males in age groups.
- Supplementary Table S6 – Distance from superior to inferior orbital rim for females and males in edentulous and dentate individuals.

Table S1. Anthropometric points used in the study.

Point	Defintion
Alare	Lateralmost located point on the edge of the *piriform* aperture[1]
Dacryon	Point on the medial border of the orbit where maxilla, frontal and lacrimal bone cross[2]
Ektokonchion	Landmark located on the lateral border of the orbit where a parallel axis to the upper margin of the orbit divides orbits in two equal halves[1]
Frontomalare orbitale	Point on the orbital rim where it meets the frontozygomatic suture [1]
Gnathion	Landmark located in the sagittal plane, at the lowest part of the lower edge of mandible[1]
Gonion	Landmark located on the intersection of the posterior border of the ramus and inferior border of the mandibular corpus[1]
Infradentale	Landmark that lies in midsagittal plane and is located anteriormost on the alveolar border of the mandible[1]
Maxillofrontale	Landmark located on the medial orbital rim, at the intersection of frontomaxillary suture and posterior lacrimal crest[1]
Nasion	Point where frontonasal suture cross with sagittal plane[2]
Nasospinale	Point located on the intersection of sagittal plane and the line drawn between most inferior points of the *piriform* aperture[2]
Orbitale	Lowest point on the lateral border of orbit[3]
Porion	Point located superiormost on the edge of the external auditory meatus[1]

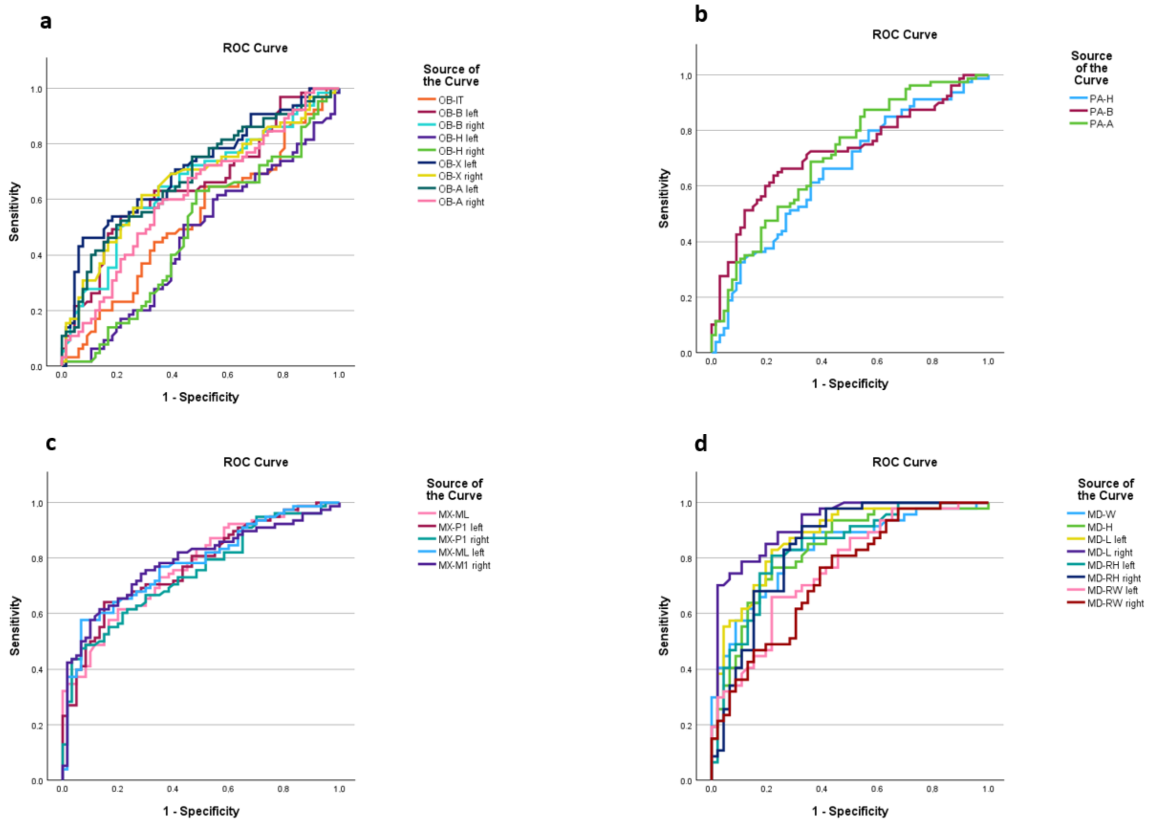

Abbreviations. Orbits: OB-IT Interorbital distance, OB-B breadth, OB-H height, OB-X X-axis, OB-A surface area. *Piriform* aperture: PA-H height, PA-B breadth, PA-A surface area. Maxilla: MX-ML height at the midline, MX-P1 height at P1, MX-MA height at M1. Mandible: MD-W width, MD-H chin height, MD-L body length, MD-RH maximum ramus height, MD-RW minimum ramus width.

Figure S2. ROC curves for facial skeleton measurements: a. orbital parameters, b. piriform aperture parameters, c. maxillary parameters d. mandibular parameters.

Table S3. Area under the ROC curves for measurements

Measurements			Area under the ROC curve
Orbit	OB-IT		0.528
	OB-B	Left	0.657
		Right	0.659
	OB-H	Left	0.461
		Right	0.484
	OB-X	Left	0.712
		Right	0.669
	OB-A	Left	0.684
		Right	0.618
Piriform aperture	PA-H		0.653
	PA-B		0.721
	PA-A		0.708
Maxilla	MX-ML		0.769
	MX-P1	Left	0.773
		Right	0.747
	MX-M1	Left	0.781
		Right	0.785
Mandible	MD-B		0.831
	MD-H		0.829
	MD-L	Left	0.872
		Right	0.921
	MD-RH	Left	0.833
		Right	0.840
	MD-RW	Left	0.762
		Right	0.736

Abbreviations. Orbits: OB-IT Interorbital distance, OB-B breadth, OB-H height, OB-X X-axis, OB-A surface area. *Piriform* aperture: PA-H height, PA-B breadth, PA-A surface area. Maxilla: MX-ML height at the midline, MX-P1 height at P1, MX-MA height at M1. Mandible: MD-W width, MD-H chin height, MD-L body length, MD-RH maximum ramus height, MD-RW minimum ramus width.

Table S4. Descriptive data of FA1 and FA2 indices for bilateral measurements.

Measurements		FA1				FA2			
		N	x̅	Min-Max	SD	N	x̅	Min-Max	SD
Orbits	OB-B	145	1.20	0.00-4.9	0.97	143	0.028	0.0002-0.12	0.02
	OB-H	149	0.64	0.01-4.00	0.63	149	0.018	0.0003-0.12	0.02
	OB-X	143	1.17	0.00-4.32	0.90	143	0.03	0.00-0.11	0.02
Maxilla	MX-P1	156	1.32	0.01-8.33	1.18	156	0.04	0.0003-0.25	0.04
	MX-M1	138	1.48	0.00-7.07	1.34	138	0.042	0.00-0.23	0.04
Mandible	MD-L	137	1.58	0.01-7.31	1.49	137	0.02	0.0001-0.09	0.02
	MD-RH	103	1.75	0.04-6.42	1.42	103	0.03	0.0007-0.12	0.03
	MD-RW	137	1.09	0.02-4.04	0.87	137	0.04	0.0007-0.17	0.03

Abbreviations. Orbits: OB-B breadth, OB-H height, OB-X X-axis. Maxilla: MX-ML height at the midline, MX-P1 height at P1, MX-MA height at M1. Mandible: MD-L body length, MD-RH maximum ramus height, MD-RW minimum ramus width.

Table S5. Distance from superior to inferior orbital rim for females and males in age groups.

Decile	Side	Females										Males									
		Young Adult			Middle Adult			Old Adult			P value	Young Adult			Middle Adult			Old Adult			P value
		N	x̄	SD	N	x̄	SD	N	x̄	SD		N	x̄	SD	N	x̄	SD	N	x̄	SD	
10	L	31	26.22	2.87	31	27.28	2.82	8	25.29	2.15	0.10	29	27.66	2.88	29	27.12	2.60	14	25.40	2.32	0.08
	R	31	**25.14**	2.14	31	26.31	2.68	7	**22.27**	1.76	0.02*	30	36.66	2.68	29	26.76	2.95	14	25.07	2.37	0.31
20	L	31	**31.19**	2.79	31	**32.54**	2.40	8	30.47	2.40	0.049*	29	32.82	2.94	29	32.70	2.24	14	31.19	2.43	0.17
	R	31	**30.83**	2.31	31	31.72	2.47	7	**28.34**	1.75	0.02*	30	31.84	2.47	29	31.85	2.37	14	31.03	1.90	0.71
30	L	31	34.36	2.58	31	35.49	2.39	8	33.63	2.52	0.08	29	35.83	2.88	29	35.83	2.12	14	34.87	2.51	0.50
	R	31	34.48	2.31	31	35.16	2.45	7	33.22	1.61	0.20	30	35.33	2.53	29	35.06	3.03	14	35.22	1.55	0.84
40	L	31	36.11	2.35	31	36.79	2.28	8	35.68	2.68	0.35	29	37.08	2.70	29	37.07	2.28	14	36.67	2.25	0.69
	R	31	36.11	2.12	31	36.81	2.48	7	35.70	1.84	0.23	30	37.02	2.30	29	36.49	2.81	14	37.41	2.08	0.48
50	L	31	36.51	2.16	31	37.15	1.91	8	37.01	2.32	0.37	29	37.35	2.50	29	37.41	2.25	14	37.26	1.90	0.64
	R	31	36.55	2.08	31	37.23	2.66	7	36.56	2.19	0.53	30	37.27	2.16	29	37.16	2.11	14	38.09	2.01	0.25
60	L	31	36.35	1.83	31	37.13	1.83	8	37.43	2.22	0.13	29	36.96	2.25	29	37.18	2.09	14	37.20	1.70	0.53
	R	31	36.18	1.82	31	37.15	2.09	7	36.45	2.51	0.19	30	36.81	1.97	29	36.95	2.06	14	37.96	1.98	0.11
70	L	31	35.48	1.71	31	36.22	1.72	8	36.68	2.24	0.14	29	35.90	2.31	29	36.12	1.83	14	36.35	1.61	0.52
	R	31	35.32	1.72	31	36.17	1.98	7	35.82	2.65	0.16	30	35.90	1.80	29	35.96	1.97	14	37.18	2.03	0.08
80	L	31	33.49	1.64	31	34.28	1.80	8	34.89	2.29	0.13	29	33.86	2.06	29	33.98	1.69	14	34.51	1.82	0.47
	R	31	33.43	1.65	31	34.25	1.92	7	34.21	2.49	0.24	30	34.05	1.76	29	34.06	1.90	14	35.34	2.14	0.13
90	L	31	29.28	1.67	31	29.75	2.05	8	30.88	2.11	0.10	29	29.78	2.05	29	29.61	1.70	14	30.87	2.04	0.15
	R	31	28.83	2.20	31	29.87	2.17	7	29.56	1.93	0.21	30	29.70	1.93	29	29.72	2.33	14	30.59	1.89	0.19

*statistically significant for $p<0.05$,Kruskal-Wallis ANOVA. Bolded values are statistically significant differences.

Abbreviations: L left, R right

Table S6. Distance from superior to inferior orbital rim for females and males in edentulous and dentate individuals.

Decile	Side	Females							Males						
		Dentate			Edentelous			P value	Dentate			Edentelous			P value
		N	x̄	SD	N	x̄	SD		N	x̄	SD	N	x̄	SD	
10	L	31	**27.28**	2.82	8	**21.29**	1.92	<0.001*	29	**27.12**	2.60	9	**22.22**	2.51	<0.001*
	R	31	**26.31**	2.68	7	**22.27**	1.76	<0.001*	29	**26.76**	2.95	8	**21.70**	3.35	<0.001*
20	L	31	**32.54**	2.40	8	**28.03**	1.51	<0.001*	29	**32.70**	2.24	9	**28.98**	2.09	<0.001*
	R	31	**31.72**	2.47	7	**28.34**	1.75	<0.001*	29	**31.85**	2.37	8	**28.65**	2.17	<0.001*
30	L	31	**35.49**	2.39	8	**31.10**	3.07	<0.001*	29	**35.83**	2.12	9	**33.25**	2.06	<0.001*
	R	31	**35.16**	2.45	7	**33.22**	1.61	0.03*	29	**35.06**	3.03	8	**32.96**	2.58	0.01*
40	L	31	36.79	2.28	8	35.16	1.88	0.07	29	**37.07**	2.28	9	**35.31**	2.04	0.04*
	R	31	36.81	2.48	7	35.70	1.84	0.21	29	36.49	2.81	8	35.13	2.63	0.08
50	L	31	37.15	1.91	8	36.73	2.16	0.64	29	37.41	2.25	9	35.88	2.38	0.07
	R	31	37.23	2.66	7	36.56	2.19	0.64	29	37.16	2.11	8	35.87	2.71	0.20
60	L	31	37.13	1.83	8	37.21	1.91	0.92	29	37.18	2.09	9	36.18	2.21	0.24
	R	31	37.15	2.09	7	36.45	2.51	0.62	29	36.95	2.06	8	36.40	2.73	0.59
70	L	31	36.22	1.72	8	36.29	1.77	0.99	29	36.12	1.83	9	35.62	2.27	0.58
	R	31	36.17	1.98	7	35.82	2.65	0.71	29	35.96	1.97	8	35.92	2.64	0.95
80	L	31	34.28	1.80	8	34.38	1.78	0.65	29	33.98	1.69	9	34.04	2.00	0.96
	R	31	34.25	1.92	7	34.21	2.49	1	29	34.06	1.90	8	34.10	2.20	0.83
90	L	31	29.75	2.05	8	29.67	1.41	0.92	29	29.61	1.70	9	29.69	2.41	0.99
	R	31	29.87	2.17	7	29.56	1.93	0.57	29	29.72	2.33	8	29.37	1.84	0.86

*statistically significant for $p<0.05$ Kruskal-Wallis ANOVA, Bolded values are statistically significant differences.
Abbreviations: L left, R right.

References

1 Nikita, E. *Osteoarchaeology: A guide to the macroscopic study of human skeletal remains*. (Academic Press, 2016).

2 Buikstra, J. E. & Ubelaker, D. H. *Standards for data collection from human skeletal remains*. (AR: Arkansas Archaeological Survey, 1994).

3 Martin, R. & Saller, K. Lehrbuch der Anthropologie. Band 2: Kraniologie. *Osteologie. Jena: Gustav Fischer Verlag* (1928).
